# Cancer prevention and screening: the next step in the era of precision medicine

**DOI:** 10.1038/s41698-018-0075-9

**Published:** 2019-01-28

**Authors:** Holli A. Loomans-Kropp, Asad Umar

**Affiliations:** 1 Cancer Prevention Fellowship Program, Division of Cancer Prevention, National Cancer Institute, Rockville, MD USA; 2 Gastrointestinal and Other Cancers Branch, Division of Cancer Prevention, National Cancer Institute, Rockville, MD USA

## Abstract

A primary mode of cancer prevention and early detection in the United States is the widespread practice of screening. Although many strategies for early detection and prevention are available, adverse outcomes, such as overdiagnosis and overtreatment, are prevalent among those utilizing these approaches. Broad use of mammography and prostate cancer screening are key examples illustrating the potential harms stemming from the detection of indolent lesions and the subsequent overtreatment. Furthermore, there are several cancers for which prevention strategies do not currently exist. Clinical and experimental evidence have expanded our understanding of cancer initiation and progression, and have instructed the development of improved, precise modes of cancer prevention and early detection. Recent cancer prevention and early detection innovations have begun moving towards the integration of molecular knowledge and risk stratification profiles to allow for a more accurate representation of at-risk individuals. The future of cancer prevention and early detection efforts should emphasize the incorporation of precision cancer prevention integration where screening and cancer prevention regimens can be matched to one’s risk of cancer due to known genomic and environmental factors.

## Introduction

Decades of basic biological and clinical research have established that a long incubation time is required for the development of malignant lesions. Even after exposure to known carcinogens, such as tobacco or human papilloma virus (HPV), cancers require substantial time to develop.^1^ Therefore, there is ample opportunity to detect early precancerous lesions and intervene during the initiation and promotion steps of the carcinogenic process, thus reversing or delaying the course of cancer progression via screening and prevention. The genomic revolution and technological advances are drivers in deciphering the molecular events contributing to disease progression and making precision targeting in cancer screening and prevention within the realm of application for benefit of high-risk individuals and then, optimistically, the general population. In this review, we will focus on the current utility of precision cancer prevention and screening strategies, as well as discuss advances enabling our ability to reduce overdiagnosis of lesions that may not ultimately progress to cancer and identify approaches to detect additional underdiagnosed lesions with a high chance of progression to cancer.

### The role of cancer prevention and early detection

Cancer is a leading cause of death in the United States, second only to heart disease.^2^ In 2018, it is estimated that ~1.7 million cancers will be diagnosed in men and women, with a corresponding 609,000 deaths cancer-related deaths.^3^ Each year, the number of incident cancer cases continues to increase globally. By 2020, the number of incident cancer cases diagnosed annually is expected to rise to 15 million.^4^ Fortunately, several cancer types, in particular colorectal, breast, and prostate cancer, can be detected by routine screening which leads to the early detection of malignant lesions.^5^

Prevention is defined as “the protection of health by personal and community-wide efforts”.^6^ These efforts are achieved by describing the burden of cancer, identifying its causes, and evaluating and implementing cancer prevention interventions.^7^ Historical perspectives of cancer prevention research have primarily focused on reducing incidence and cancer-related mortality. Early efforts in cancer prevention focused on both synthesized chemicals (e.g. retinoids, tamoxifen, etc.) and natural compounds (e.g. β-carotene, omega-3 fish oil, etc.).^8^ Efforts have more recently broadened to include interventions focused on ‘pre-disease’ or those intended to delay carcinogenesis.^9,10^ These tasks, however, are easier said than done. National- and global-level organizations, such as the National Cancer Institute (NCI), the World Health Organization (WHO), and the International Agency for Research on Cancer (IARC), have the resources and capabilities to accurately represent the population-level burden of cancer. However, prevention initiatives should first have substantial impact on the individual-level to ultimately translate to a population-level benefit, implying the view that population health is the collective health experience of individuals.^11^

**Fig. 1:**
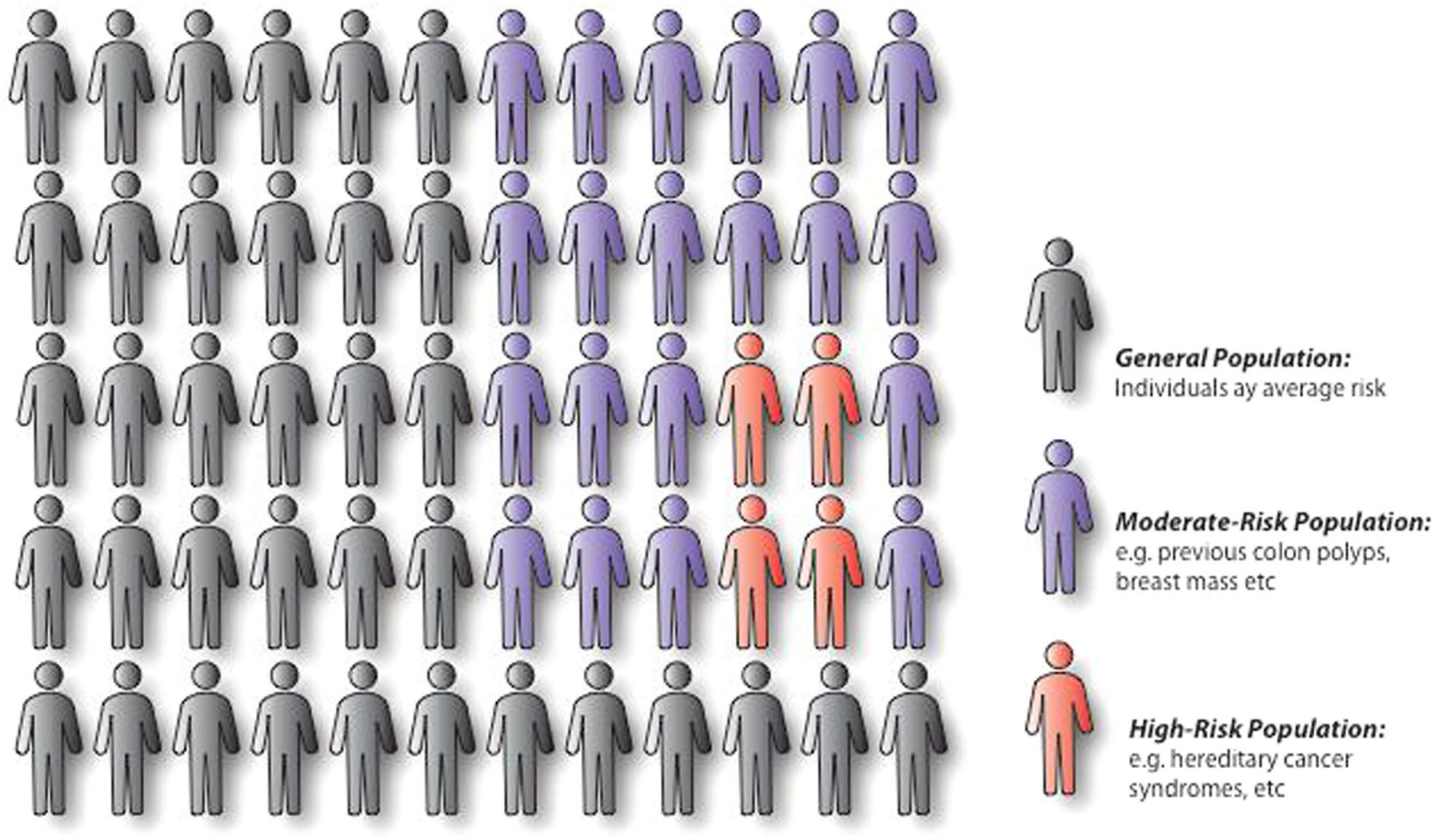
Cancer risk varies within a population. Individuals within a population will have differing baseline levels of cancer risk, which may be influenced by genetic and environmental factors, or the interaction of genetic and environmental factors. The general population (black) is considered individuals of average cancer risk. Over time, one’s risk may increase as a consequence of age or exposure (purple). However, an individual may be considered high-risk if, despite age or exposure, he or she has a heritable condition (e.g. Lynch syndrome), a family history of cancer suggesting genetic susceptibility, or a personal history of cancer (red). The high-risk population may also incur increased risk over time due to age and exposure

Cancer risk is influenced by a mixture of genetic and environmental factors, such as behavioral, lifestyle, and environmental exposures (Fig. 1). An individuals’ risk is the sum of these various factors, though the effect magnitude of a single factor is difficult to quantify. In vitro and in vivo experimental analysis has allowed for the identification of genes, such as *FOXA2*, *PIK3CA*, and *RB1*, which can drive cancer initiation and progression.^12^ This process becomes more complex with the addition of new genetic events.^13^ These complicated genetic signatures may be further influenced by environmental exposures. Population attributable fraction (PAF) is a measurement intended to better define the disease-risk of an individual environmental exposure. For example, a study conducted in the United Kingdom estimated a PAF of 19.4% for tobacco and 3.7% for occupational exposures, contributing to ~60,800 and 11,500 cancer cases annually, respectively.^14^ However, various factors associated with one’s risk of cancer, such as length of time exposed, exposure level, and exposure sum, are not accounted for in this calculation. Therefore, determining the true impact of individual factors on ones’ overall cancer risk is problematic to ascertain.

**Fig. 2:**
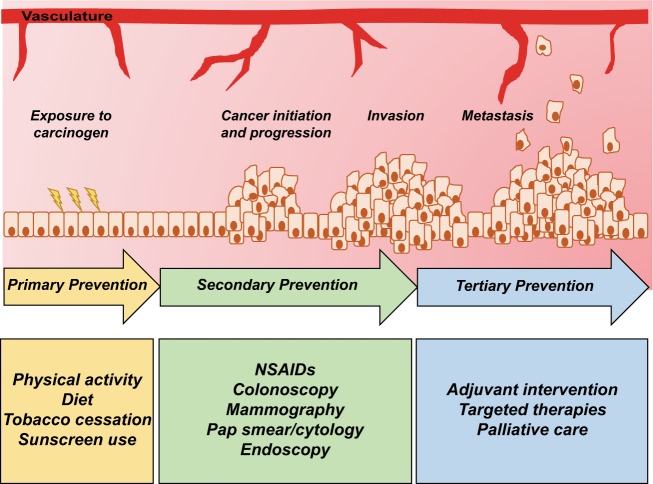
Cancer prevention can occur throughout the cancer initiation and progression spectrum. Prevention strategies may be efficacious throughout the span of cancer initiation and progression. Primary prevention mechanisms, which include alterations in physical activity or diet, tobacco cessation, or use of sunscreen, may reduce the impact of exposures in cancer initiation. Secondary prevention strategies, such as cancer screening or use of non-steroidal anti-inflammatory drugs (NSAIDs), allow for the early detection of precancerous lesions or help inhibit progression to cancer. Tertiary prevention methods, including chemotherapy or targeted therapy, may be used to help keep a localized cancer from spreading or metastasizing

Several prevention and early detection mechanisms have been identified to aid in reducing cancer incidence and are multilevel, involving primary, secondary, and tertiary processes (Fig. 2). Primary cancer prevention involves the direct avoidance or reduction in exposure to known carcinogenic factors.^6^ Key examples of primary prevention include tobacco cessation, changes in diet (e.g. decreased red meat consumption, limiting fatty foods) and increased physical activity.^6,7^ Primary prevention methods involve modifying lifestyle factors that confer risk of developing cancer (e.g., exercise, tobacco cessation, and nutritional supplements) and protective therapeutics (e.g. vaccination) which have demonstrated long-term efficacy for cancer prevention.^15,16^ Secondary prevention helps to stagnate, inhibit, or reverse carcinogenesis. These methods often involve the early detection, treatment, or removal of precancerous lesions, which will be further defined in the next section.^16,17^ For example, colorectal adenomas or early-stage colorectal cancers can be identified through screening by colonoscopy, a secondary prevention modality. Additionally, testing for HPV DNA or co-testing with the cytology-based Pap smear can detect cervical cancer-associated HPV infections.^18^ Current recommended strategies for primary and secondary prevention for various cancer types are detailed in Table 1. Tertiary prevention can be initiated after a diagnosis of cancer to improve quality of life and survivorship.^17^ It is important to note that the definitions of primary, secondary, and tertiary prevention can vary, however the overall message of prevention is the same. Being aware of the complexities of individual and population cancer risk and being armed with a multitude of prevention strategies puts the health care community in a prime position for cancer prevention.

**Table 1:** Examples of currently recommended and possible future precision cancer prevention strategies for selected cancers in the United States

Cancer	Primary prevention strategies	Secondary prevention strategies	Precision prevention strategies
Colon	Adults aged 50–59 years with an increased 10-year risk of cardiovascular disease are recommended to take low-dose aspirin daily for at least 10 years^85^	Individuals between the ages of 50 to 75 should receive a colonoscopy every 10 years, sigmoidoscopy with every 5 years with fecal occult blood test every 3 years or annual fecal immunochemical test ^63^	Colonoscopy should be performed based on individual risk and/or genetic information (e.g. family history, Lynch syndrome, and mutations in colorectal cancer-specific genes)
Breast	For women with an increased risk of breast cancer, an individual decision to take risk-reducing medications, such as tamoxifen or raloxifene should be made^86^	Women between the ages of 50–74 should receive a biennial mammography^87^	Screening recommendations based on individual risk factors, such as breast density, family history, genetic information (mutations in breast cancer-specific genes), or exposures (e.g. history of chest radiation)^87^
Prostate		Men age 55–69 years should make an individual decision to undergo prostate-specific antigen (PSA)-based screening^88^	Mutations in prostate cancer-specific genes
Lung	Abstinence from tobacco products, reduce exposure to asbestos or radon^89^	Low-dose computed tomography for adults aged 55–80 who have a 30 pack-year smoking history and currently smoke or a former smokers in the last 15 years^90,91^	Identification of molecular markers of cancer risk and can predict indolent versus aggressive lesions^92^
Skin	Young adults, adolescents, children, and parents should be counseled about minimizing exposure to UV radiation^93^	Screening by physical exam checking for moles, birthmarks, or other pigmented areas	Panel testing for common genetic mutations
Cervical		Women age 21–29 years should be screened by cervical cytology every 3 years, while women age 30–65 years should be screened by cervical cytology every 3 years, HPV testing every 5 years, or cytology/HPV co-testing every 5 years^94^	Cervical cancer screening (HPV-test) should be performed based on individual risk influenced by lifestyle factors or exposure and/or genetic information

### The case for the early detection of cancer

Multistep tumorigenesis is required for the transformation of a normal cell to a cancerous cell. Boland and Ricardo provide a simplified model of tumorigenesis: an organism accumulates a number of genetic mutations due to exposures or errors during DNA synthesis or mitosis, which leads to the aberrant growth of a cell through a series of truncating or missense mutations and genetic deletions. Though most errors occurring during replication are silent, nonfunctional, corrected, or result in cell death, occasionally a gene becomes mutated, primarily through a combination of frameshifts (insertion/deletions) and missense mutations that impact the functionality of the cell.^19,20^ Successive somatic genomic or epigenomic alterations may provide additional cellular advantages that lead to invasive cancer. Data from a comprehensive examination of molecular cancer subtypes indicated that 5–10 genetic alterations are required to induce a malignant phenotype. Studies in colorectal cancer have demonstrated that at least seven genetic events are necessary for transformation.^21^ In some hereditary and sporadic cancers, genomic instability appears to occur in the early cancer stages with progressive destabilization over time.^22^

Often the pathways altered during early tumor development impact specific physiological processes, such as tissue repair, wound healing, vascularization, or the co-option of surrounding cells through direct contact, secreted growth factors, or gene transfer by extracellular vesicle secretion.^23,24^ The somatic mutation theory suggests that stochastic somatic mutations result in the selection of genetic alterations that provide advantage for uncontrolled proliferation and tumorigenesis.^25,26^ However, this theory has not been scientifically tested and has several shortcomings, including no mention of methods which can induce DNA alterations without mutation, such as epigenetic alterations, or the inclusion of the tumor microenvironment (the effect of the stroma and immune milieu). Experimental evidence has suggested that either global genetic instability, such as chromosomal or microsatellite instability from issues during cell cycle or DNA repair (e.g., mismatch repair) deficiency, is needed for the progression of most cancers.^20^ The ‘cooperative’ tissue microenvironment induced by the cancer cells or as part of a premalignant niche is necessary for tumor expansion and results in detectable changes in the tissue. Models of pancreatic cancer have demonstrated the necessity of fibroblasts in tumor cell propagation, while additional carcinogenesis models have shown that recruitment of endothelial cells to the tumor is necessary for growth beyond 1–2 mm^3^.^27,28^

Cancer progresses in spatial and temporal domains, where it may take years to decades for normal cells to transition into invasive cancers; in addition, multiple cell clones within an individual or within the same cancer types in others can evolutionarily diverge to present as separate entities. Hence, the molecular aberrations that initially lead to transformation continue their evolution and further develop into heterogenous cancers. Premalignancies exhibit varying degrees of disparate genetic, molecular, and transcriptomic profiles compared to both normal tissue and malignant tumors, however, some data suggests that this is dependent on invasive potential. Specific examples from breast and esophageal cancers accentuate the necessity of understanding this variability. Studies utilizing breast cell lines have shown distinct expression signatures occurring along the spectrum of normal to invasive carcinomas; many of the identified modifications were epigenetic.^29^ Furthermore, the transcriptomic profiles of women were substantially different between those who developed breast cancer up to 5-years before diagnosis and women who were cancer-free, suggesting that many of these alterations occur prior to tumor initiation and priming of the malignant niche.^30,31^ Similar patterns of genetic alterations have been observed in Barrett’s esophagus, a known precursor lesion to esophageal adenocarcinoma, and colorectal adenomas, which can transform into adenocarcinomas. Non-dysplastic Barrett’s esophagus has a lower mutational burden (5.4–6.8 single nucleotide variations per megabase) compared to dysplastic Barrett’s esophagus, which, in turn, has a lower mutational burden than esophageal adenocarcinoma.^32,33^ Additionally, several mutations have been mapped across the normal to malignant spectrum. Recurrent mutations in *TP53* have been observed in high-grade dysplastic Barrett’s esophagus and esophageal adenocarcinoma, however *SMAD4* mutations have only been observed in invasive lesions.^32^ The differential profiles of cancer through tumorigenesis starting at early lesions to advanced/invasive disease may provide a unique avenue for early detection, particularly if we can pinpoint specifically which early events leads to cancer progression. However, molecular profiling of early lesions can be an expensive enterprise as a clear majority of premalignant lesions do not progress to invasive cancer and an increasingly larger sample size is required to identify drivers of progression as we move toward early precancerous lesions.

Early cancer detection modalities, such as mammography, colonoscopy, prostate-specific antigen (PSA) screening, and cervical cell cytology (Pap smear), have been widely implemented. Mammography is an accepted practice with relatively high adherence in areas where resources are available. Though mammography is considered the ‘gold standard’ for breast cancer early detection at a curable stage, it is not without its costs. False positives, which leads to unnecessary patient biopsies, and overdiagnosis, which is the detection of lesions that would ultimately not lead to a cancer-related death, occur at a high rate.^34^ Similarly, routine PSA testing for the detection of prostate cancer in the United States has resulted in a similar outcome, causing unnecessary follow-up and overtreatment. Great Britain, which does not habitually use PSA for screening, has comparable prostate cancer rates to the United States, questioning the utility of PSA for the early detection of prostate cancer.^10^

Despite issues, there have been several triumphs in cancer screening. A major success was the implementation and dissemination of cervical cancer cytology screening, or Pap smears. This minimally invasive screening procedure allows for the direct visualization of cellular changes occurring in the cervix, which can then be used to appropriately inform treatment options such as colposcopy or cryotherapy. The results of Pap smears alone, however, can be detrimental. A modeling study investigating cervical cancer outcomes in the Dutch registry estimated that approximately 74% of cervical cancers were a result of overdiagnosis.^35^ When paired with HPV testing, the overall outcomes improved.^36^ Therefore, when implementing and evaluating cancer prevention strategies, it is necessary to consider and optimize the risk-benefit ratio of the methodology and limit the possibility of undue harm to the patient population.^37^

### The case for precision cancer prevention

A substantial body of research has been devoted to investigating the pathways involved in cancer initiation, progression, and metastasis, and identifying biomarkers associated with these mechanisms for clinical utility. The term ‘biomarker’, however, is broad and encompasses a multitude of biological features or molecules, such as imaging or radiomic alterations, DNA modifications, expression of different RNA types, and metabolomic and proteomic changes. Regardless of the biomarker type, the ideal biomarker should be: (1) directly associated with the disease of interest, (2) be involved in at least one part of the cancer spectrum, (3) provide high sensitivity and specificity, (4) have relatively non-invasive detection, and 5) have a reasonable cost-benefit ratio.^38^ The success of individual biomarkers has been limited, however progress has been made in the use of biomarker panels for the early detection and precision treatment of several cancers, such as breast cancer and melanoma. The American Society for Clinical Oncology recommends tumor-typing for estrogen receptor (ER), progesterone receptor (PR), and human epidermal growth factor receptor 2 (HER2) in breast biopsies. The information attained from this tumor-typing can then be used to inform interventive options, such as tamoxifen for ER-positive cancers, trastuzumab for HER2-positive cancers, or the need for additional genetic analysis^.^^39,40^ A similar tiered approach can be utilized in the early detection of melanoma harboring BRAF-V600E mutations. Approximately 50% of melanomas have a mutation in BRAF which results in the constitutive activation of the Ras/Raf/MEK pathway. Of these, over 90% of the BRAF mutations are a missense mutation resulting in the substitution of valine for glutamic acid at codon 600.^41^ Early detection of melanoma and tumor-typing for the BRAF-V600E mutation can then inform therapeutic options, such as treatment with vemurafenib or dabrafenib which specifically target the activating mutation.^42^

Ideally, however, biomarkers should be used to determine the potential effectiveness of a cancer prevention modality, rather than waiting until presentation and diagnosis. One such example is the use of aspirin for the prevention of colorectal cancer. Though the use of low-dose daily aspirin is generally recommended for older individuals (age 50–69) with specific cardiovascular disease risk, aspirin use has demonstrated increased efficacy for colorectal cancer prevention in similar age group individuals with distinct biomarkers.^43–45^ For example, a nested case-control analysis of the Nurses’ Health Study and the Health Professional Follow-up Study, two large prospective cohort studies, found that individuals with the single nucleotide polymorphism (SNP) rs6983267 T allele in *CTNNB1*, which codes for the protein β-catenin, influences the formation of the Wnt/β-catenin destruction complex. Aspirin reduced β-catenin expression, which in turn inhibits transcription and activation of MYC, reducing colorectal cancer potential.^46^ On the other hand, at the same location in *CTNNB1* the substitution to a G allele, rather than a T allele, results in increased destruction complex binding and may promote colorectal cancer progression.^47^ Similar associations of reduced colorectal cancer risk have been observed for the SNPs rs2965667 (TT) in *MGST1* and rs16973225 (AA) located near *IL16*.^47^

Unfortunately, the integration of such molecular mechanisms for the early detection and treatment of cancer is not widespread, though genomic classification of various cancers may lead to improvements in cancer prevention strategies and help identify high-risk individuals.^7^ Particularly, genomic characterization of cancer may pinpoint actionable alterations which can then be used for targeted interventions.^48^ Screening recommendations for colorectal cancer have revolved around a similar strategy. Current screening guidelines for colorectal cancer stratify individuals into four risk groups: (1) a high-risk group, which includes hereditary conditions such as familial adenomatous polyposis and Lynch syndrome, (2) a moderate-risk group, which includes acquired increased-risk conditions, such as inflammatory bowel disease or colitis, (3) an average risk group (i.e. individuals over the age of 45 or 50), and (4) a low risk group that excludes the other outlined groups.^38,49,50^ Despite stratification, there remains intergroup heterogeneity, referring to different levels of individual disease risk within a stratified group, which may be approached by further stratification of risk factors within each group. Investigation of a risk index for the development of advanced neoplasia in average-risk individuals using lifestyle and behavioral factors, such as body mass index (BMI) and smoking status, can identify low- and high-risk individuals within this group. This information may be further used to advise screening strategies.^51^

With this approach in mind, the Women Informed to Screen Depending on Measures of Risk (WISDOM) study was designed to pragmatically investigate annual versus risk-based screening for breast cancer.^52,53^ Only 10% of women have an accurate perception of their risk for breast cancer and 40% of women have never discussed their risk of breast cancer with their doctor.^52,54^ The WISDOM trial seeks to establish the efficacy of risk-based screening, which integrates clinical risk factors (e.g. BMI, age), breast density, and polygenic risk score, compared to annual screening for breast cancer, which will be determined through the number of biopsies performed and measurement of participant morbidity.^52,53^ Though the completion and analysis of the WISDOM trial is far from finished, this study may move the needle to begin integrating precision risk-based screening strategies for certain cancers.

Rapid scientific and technological advances should allow for a more accurate stratification of at-risk individuals who may benefit from prevention strategies. This may include increased screening or surveillance, alternative treatments, or early aggressive treatment.^55^ This method may also involve decreased screening or surveillance for low-risk groups. A working group investigating the utility of precision risk stratification-based screening and recommendations for colorectal cancer from United States Military Health System suggested excluding low-risk individuals from screening who would likely be cured by surgical intervention alone, identify and locally treat patients with early stage CRC, and limit treatments to patients with latent or stable disease.^55^

### Precision prevention

As the idealistic goal of cancer prevention is to inhibit the formation of cancer prior to initiation, primary prevention is critical to accomplish this goal. Precision prevention incorporates precision medicine approaches and the individuals’ risk profile, which is defined by genomic and lifestyle risk factors.^7,56^ The current age of scientific innovation has identified several techniques that may be fortuitous, primarily in early detection of cancers and exploit genetic risk factors. For instance, circulating cell-free DNA (cfDNA) can be isolated from plasma, serum, urine, or other body fluids as a surrogate biomarker (i.e. m*SEPT9*, *KRAS*, and *CDKN2A*), to examine overall disease burden, or perform Next-generation sequencing to develop genetic profiles.^57^ Generally, elevated levels of cfDNA and circulating tumor DNA (ctDNA) can be detected systemically in patients with cancer, however cfDNA is not specific to cancer and may be linked with other pathologies.^38^ On the other hand, as cfDNA can be sequenced, the discovery of genetic aberrations, such as mutations (e.g., in *KRAS, TP53*, or *APC* or frameshifts in microsatellites) may provide a more precise tool to identify the presence of early or previously undetected cancer.^38,48^ Similarly, ctDNA and circulating tumor cells (CTCs) can be isolated from plasma or serum and assessed for molecular alterations.^48^ For example, Morelli and colleagues examined pre- and post-treatment plasma from patients with metastatic colorectal cancer and showed detectable *EGFR* and *KRAS* mutations. Detection of *EGFR* after treatment correlated with lower progression-free survival.^58^ However, the detection of ctDNA and CTCs is currently not reliable for detection of either early-stage disease due to a paucity of tumor cells in precancers; nonetheless, this technique has demonstrated some utility in predicting risk of recurrence after treatment.^55,59^

The usability of cfDNA, ctDNA, and CTCs for the early detection of cancer has promise, yet there are several limitations to these techniques. The described methods have little effectiveness in detecting premalignant or early stage disease due to low systemic abundance of the marker and limitations in assay sensitivity and reproducibility.^48,55^ Other issues, such as varying expression between individuals, may impact the reliability of tumor biomarkers. The biomarker of interest may not be specific to tumor cells and produced by normal or non-cancerous cells, may not be produced by early lesions, or may not be produced by all tumor cells.^60^ Therefore, there are several scenarios in which early lesions may be missed by screening. Furthermore, as with all new technologies, implementation of these detection modalities is currently not feasible on a large scale. Most clinical centers do not have the resources available to perform the assays necessary for the evaluation of cfDNA, ctDNA, or CTCs. Not only can these assays be experimentally challenging and exhibit high variability, for the accurate early detection of cancer we must also know the underlying molecular aberrations of the disease for detection. Though there are high frequency genetic changes that occur early in carcinogenesis for particular cancers, the genomic landscape of cancer is vast.^21^ The unique genetics of an individuals’ tumor complicates our ability to develop modalities to properly identify profiles.^21^ With the ongoing development of the Human Tumor Atlas and the Precancer Atlas, the knowledge of these disease states and potential genetic targets is expanding but is currently outside of population-level feasibility.

### The future of precision screening and prevention

We must first ask: is cancer prevention necessarily precise? The next logical question is: does it matter? Several recommended cancer prevention strategies that have demonstrated substantial efficacy tip-toe around the idea of precision. Long-term use of non-steroidal anti-inflammatory drugs (NSAIDs) and cyclooxygenase-2 inhibitors (COXIBs) have been associated with a reduced risk of developing several gastrointestinal cancers, though an inhibitory effect has been observed consistently in colorectal cancer.^37,61^ NSAID and COXIB use has been effective in reducing incidence of adenomas and colorectal cancer in both hereditary/high-risk and average-risk populations.^62^ The United States Preventative Services Task Force (USPSTF) has integrated a precision prevention approach into population-level evidence-based recommendations. For individuals ages 50–59 with a long-term (>10 year) risk of cardiovascular disease and a life-expectancy of at least 10 years who are willing to take low-dose aspirin daily and are not at risk of bleeding, the USPSTF recommends the use of low-dose aspirin for the prevention of both cardiovascular disease and colorectal cancer. This recommendation changes for individuals between the ages of 60–69, such that the decision should be personal. Similarly, for those above 69 years old, there is no recommendation to use aspirin as the data is currently lacking. The overall guidelines (e.g. long-term risk of cardiovascular disease, not at increased risk of bleeding) remain the same, however the decision to initiate daily low-dose aspirin should occur on an individual basis.^63^

NSAIDs and COXIBs have shown reasonable efficacy as cancer prevention agents for gastrointestinal cancers, but can this be considered precision prevention? NSAIDs inhibit cyclooxygenase (COX)−1 and COX-2, reducing prostaglandin biosynthesis and inflammation.^37^ COXIBs act through a similar mechanism and specifically target COX-2.^64,65^ Therefore, use of NSAIDs and COXIBs do not just reduce gastrointestinal inflammation, but systemic inflammation. Perhaps the initiation and progression of gastrointestinal cancers is more dependent on persistent inflammation compared to other primary tumor sites. This can be inferred from the association between chronic inflammatory diseases, such as inflammatory bowel disease and Crohn’s disease, and increased risk for colorectal cancer.^66,67^ Obesity is also associated with chronic inflammation, which may play a role in the efficacy of aspirin in preventing colorectal cancer. Rothwell and colleagues recently investigated the effect of aspirin on the risk of cardiovascular events and cancer in a collective of randomized trials and found that the preventative effect of aspirin on cancer risk is dependent upon bodyweight and dose. If this can be confirmed in subsequent studies, this observation calls for a personalized dose and/or regimen of aspirin and probably other cancer preventives based on body weight.^68^ Similar associations have been observed between *Helicobactor pylori* infection and gastric cancer, esophagitis and esophageal adenocarcinoma, and pancreatitis and pancreatic cancer.^69^ NSAIDs and COXIBs function to precisely suppress inflammation, yet the impact is systemic and not ultimately intended for the specific risk reduction of gastrointestinal cancers.

The association between NSAID or COXIB use and reduced risk of gastrointestinal cancers, but not other cancer types, highlights an important detail that significantly impacts precision prevention: tumor heterogeneity. In this case, tumor heterogeneity not only refers to variations in genetic and molecular profiles between and within tumor types, but also the behavioral and environmental factors and the biological alterations that guide initiation and progression of malignancies. Some cancer prevention agents can be more strictly classified as a precision prevention agent. Tamoxifen and raloxifene are selective estrogen receptor modulators (SERMs) and have demonstrated significant efficacy in the prevention of ER-positive breast cancers.^70,71^ However, because these agents, as well as aromatase inhibitors, target hormone receptors which are organ-specific, their effectiveness is limited to cancers that express ER and are not particularly beneficial against the other breast cancer subtypes.^71^ The Breast Cancer Prevention Trial P-1, Multiple Outcomes of Raloxifene Evaluation, and Study of Tamoxifen and Raloxifene (STAR) trials investigated the effectiveness of SERMs to prevent the development of breast cancer in at-risk women (e.g. post-menopausal, over 35 years old). These trials demonstrated a reduction of invasive breast cancer incidence by 49%, 70%, and 44–90%, respectively.^72–75^ However, the STAR trial showed an increased risk of uterine cancer among individuals given tamoxifen, thus indicating a potential harm of SERMs for prevention.^75^ To potentially counteract the detrimental effect of systemic SERMs, a current breast cancer prevention trial is investigating the efficacy of topical application of tamoxifen, allowing for localized prevention (NCT02993159).

A similar argument can be made for other cancer prevention agents with particular molecular targets, such as the epidermal growth factor receptor (EGFR) inhibitor erlotinib for the prevention of head and neck cancer.^76^ Erlotinib, in combination with celecoxib, was used in a Phase Ib/pharmacokinetic study of head and neck cancer prevention among individuals with oral leukoplakia, dysplasia, and carcinoma in situ. Though the trial demonstrated initial efficacy, majority of the premalignant lesions recurred or progressed.^77^ The Erlotinib Prevention of Oral Cancer (EPOC) trial, a randomized, placebo-controlled, double-blind trial which used loss of heterozygosity and *EGFR* copy number to stratify and evaluate patients, also lacked the clinical evidence to support the use of erlotinib for head and neck cancer prevention, as *EGFR* expression was unable to predict erlotinib efficacy.^78^ Unless the overexpression, activation, or mutation of the molecular target is consistently constitutively expressed across and within cancer types and is necessary for carcinogenesis, universal cancer prevention with a single agent is a difficult hurdle to overcome. Even if the marker is constitutively expressed, there is no guarantee that the targeted agent will prove efficacious. Furthermore, with the addition of more agents needed to achieve preventative value through synergistic action, the risk of unacceptable toxicities increases, particularly for agents requiring long-term dosing.

In cancer prevention, does precision matter? Sometimes. When we can tie specific genetic factors, such as epigenetic or genetic alterations, and other non-genetic factors, such as family history, to accurately assess ones’ risk of a particular type or subset of cancers, precision screening, surveillance and prevention is a useful approach as we can adjust risk-benefit profiles based on the risk of invasive cancer. Conversely, for much of the population who are individuals at average-risk for cancer development, utilizing the more global cancer preventative measures, such as NSAIDs, adjusting lifestyle factors (e.g. diet, exercise, and tobacco use), and screening, may ultimately prove more beneficial. Therefore, we need to better identify a stratification strategy where the risk-benefit ratio is favorable in individuals with an increased risk of cancer or who have an increased risk of exposures to cancer-causing substances, and that individuals with low risk incur minimal harm. Though generalized anti-cancer initiatives are not precise, these methods can reach a larger population and expand their impact. Assessing and identifying the impact of different cancer types will provide information on prioritizing cancer prevention initiatives and strategies, whether those strategies are based at the individual- or population-level.^79^

## Conclusion

The goal of early detection and prevention of cancer is to reduce, reverse or eliminate ones’ risk of developing and dying of cancer. To do so, we must comprehend and assess cancer as a pathology and as a system. This requires understanding the population- and risk-based associations with cancer (behavior, socioeconomic factors, and epidemiology) and the fundamental mechanisms of tumorigenesis (genetics, epigenetics, signaling, tumor microenvironment, and immune factors), and integrating this knowledge in a tangible manner.^17,80^ A key illustration integrating gained knowledge to improve diagnosis to guide precision therapy is the development of genetic tests, such as MammaPrint for breast cancer, Therascreen^®^ for lung cancer, and cobas 4800 BRAF V600 for melanoma, to identify mutations and alterations in the bulk tumor.^81^ However, having these tools available and implementing the widespread use of these tools for diagnosis and therapy can be difficult. Though the field of oncology has progressed substantially in precision medicine initiatives in diagnosis and therapy, cancer screening and prevention has not caught up to those advances. Nonetheless, cancer prevention and early detection could, and perhaps should, adapt the techniques that are now commonplace in precision medicine to precision prevention initiatives. The use of cancer immunoprevention and immunotherapy has already begun moving in the precision prevention direction. Vaccines targeted against viruses such as hepatitis B virus and HPV, or tumor antigens, such as MUC1, have demonstrated efficacy in the prevention of hepatocellular carcinoma, cervical cancer, and models of colorectal cancer.^82^ The PATRICIA trial of HPV presented stunning results with over 90% vaccine efficacy for lesions with HPV-16 or HPV-18 infection.^83^ Early phase treatment with a MUC1 vaccine have illustrated enhanced immune response to presenting MUC1 tumor antigens.^84^ However, the use of vaccines for cancer prevention may not be applicable for all cancer types. Therefore, though there will likely never be a single, unequivocal strategy for cancer prevention, the integration for what is known and what has yet to be uncovered will direct the future of the field.
